# Robot-assisted arthroscopic all-epiphyseal PCLR with remnant preservation in a 13-year-old boy: a case report and review of the literature

**DOI:** 10.3389/fsurg.2025.1595715

**Published:** 2025-06-26

**Authors:** Chaofan Liao, Jiang Zheng, Qiuzhen Liang, Peidong Liu, Panpan Pang, Liang Zhang

**Affiliations:** ^1^ Graduate School, Xi'an Medical University, Xi'an, China; ^2^ Sports Medicine Center, Honghui Hospital, Xi'an Jiaotong University, Xi'an, China

**Keywords:** robotics, adolescent, PCLR, all-epiphyseal, case report

## Abstract

**Purpose:**

There is limited research worldwide on posterior cruciate ligament (PCL) tears in pediatric and adolescent patients (PAPs). This report aims to present our treatment method as a potential reference for clinical surgery.

**Methods:**

We report the case of a 13-year-old boy with a PCL tear who underwent robot-assisted arthroscopic all-epiphyseal PCL reconstruction (PCLR) with remnant preservation using the TiRobot surgical robot. The patient was followed for 6 months postoperatively.

**Results:**

The surgery lasted 110 min, involving four x-ray exposures and a single guide pin insertion, without requiring positional adjustment. Postoperative magnetic resonance imaging on day 2 confirmed that the femoral and tibial bone tunnels were within the epiphysis, with good graft fixation. The angle between the reconstructed tibial bone tunnel and the graft was approximately 104.1°. Sutures were removed after 2 weeks, showing good wound healing and full extension of the affected limb. By 8 weeks, the patient had regained full knee flexion, and by 12 weeks, muscle strength of the affected limb exceeded 85% of that in the contralateral side, allowing the patient to start jogging. By 16 weeks, the patient resumed badminton training. At the last follow-up, knee function had markedly improved, with the preoperative International Knee Documentation Committee score increasing from 43.68 to 82.76 and the Lysholm score increasing from 46 to 95.

**Conclusion:**

Arthroscopic all-epiphyseal PCLR with remnant preservation, assisted by the TiRobot orthopedic robot navigation system, demonstrated several clinical advantages. The technique theoretically avoids damage to the PAPs’ growth plate, preserves the PCL remnants, reduces the “killer turn” effect, and minimizes the risk of injury to surrounding blood vessels and nerves. Serial radiographic evaluations during the 6-month follow-up revealed no evidence of physeal damage in this case.

## Introduction

In the discourse surrounding knee injuries, the posterior cruciate ligament (PCL) has often received less attention despite being a crucial component, accounting for approximately 1%–44% of such injuries (1). Compared to anterior cruciate ligament (ACL) injuries, the incidence of PCL injuries is indeed lower. This is primarily because the ACL is not as robust as the PCL in terms of anatomical structure and biomechanical properties. In addition, the ACL bears a greater load during movement and plays a more prominent role in dynamic stabilization during knee joint activities, making it more susceptible to injury (2). Nonetheless, PCL injuries should not be underestimated, particularly in pediatric and adolescent patients (PAPs). Although the incidence is relatively low in this population, diagnosis poses significant challenges due to the unique characteristics of bone development in PAPs and the relatively concealed anatomical position of the PCL.

The causes of PCL injuries in PAPs are diverse, with sports injuries being the most common, accounting for approximately 38.8% of cases (3). This is particularly true in high-intensity sports such as basketball, soccer, and skiing, where sudden directional changes, jumping, or unstable landings can exert significant stress on the PCL. In addition, “dashboard injuries” resulting from car accidents represent another major cause of PCL injuries. During a collision, external force impacting the occupant's knee can directly impact the PCL, leading to injury. Moreover, these young patients often cannot accurately describe their symptoms, and the clinical presentation of PCL injuries may not be as obvious as ACL injuries, resulting in frequent cases of missed or incorrect diagnoses.

Currently, there is a notable paucity of literature regarding treatment modalities for PCL injuries in PAPs, and consensus on the optimal approach remains elusive. Decisions concerning whether to perform a surgical intervention, the timing of such an intervention, and the choice of surgical technique continue to be subjects of considerable debate. On the one hand, conservative management may result in further irreversible meniscal damage, accelerated cartilage degradation, and a marked reduction in overall knee stability (4). On the other hand, surgical repair of the PCL that traverses the growth plate carries the potential risks of angular deformities and growth arrest in PAPs (2). In current clinical practice, conservative treatment is often favored as the initial approach for PAPs with PCL injuries due to their unique bone growth characteristics (5). Nevertheless, with advances in medical technology, an increasing number of scholars report that employing advanced epiphysis-protecting surgical techniques does not negatively impact the growth and development of such adolescent patients (1, 6–9).

In recent years, the integration of surgical robotics with arthroscopic techniques has reached a significant level of maturity. Zhang et al. (10) documented the utilization of robot-assisted navigation in all-epiphyseal ACL reconstruction (ACLR), highlighting its primary advantage: the precise localization of bone tunnels with minimal error margins, thereby theoretically minimizing the risk of growth plate injury. We considered that for PAPs presenting with open growth plate PCL injuries, robotic technology holds substantial promise in enhancing surgical outcomes.

## Case presentation

### Patient profile

A 13-year-old boy presented with pain and swelling in the right knee joint that persisted for 3 days following an injury sustained during a bicycle accident (Table 1).

**Table 1:** Basic patient information.

Patient information	Situation
General information	
Sex	Male
Age	13 years
Height	168 cm
Weight	64 kg
Knee	Right
Cause of injury	Bicycle accident
Physical examination	
Step-off sign	(+)
Posterior	(+)
Posterior drawer test	(+)
Anti-Lachman test	(+)
Anterior drawer test	(−)
Valgus stress test	(−)
McMurray test	(−)
Laboratory examination	
ALP	256.00 U/L
Urine pH	5
Cr	39.00 μmol/L

ALP, alkaline phosphatase; Cr, creatinine.

### Preoperative evaluation

**Figure 1:**
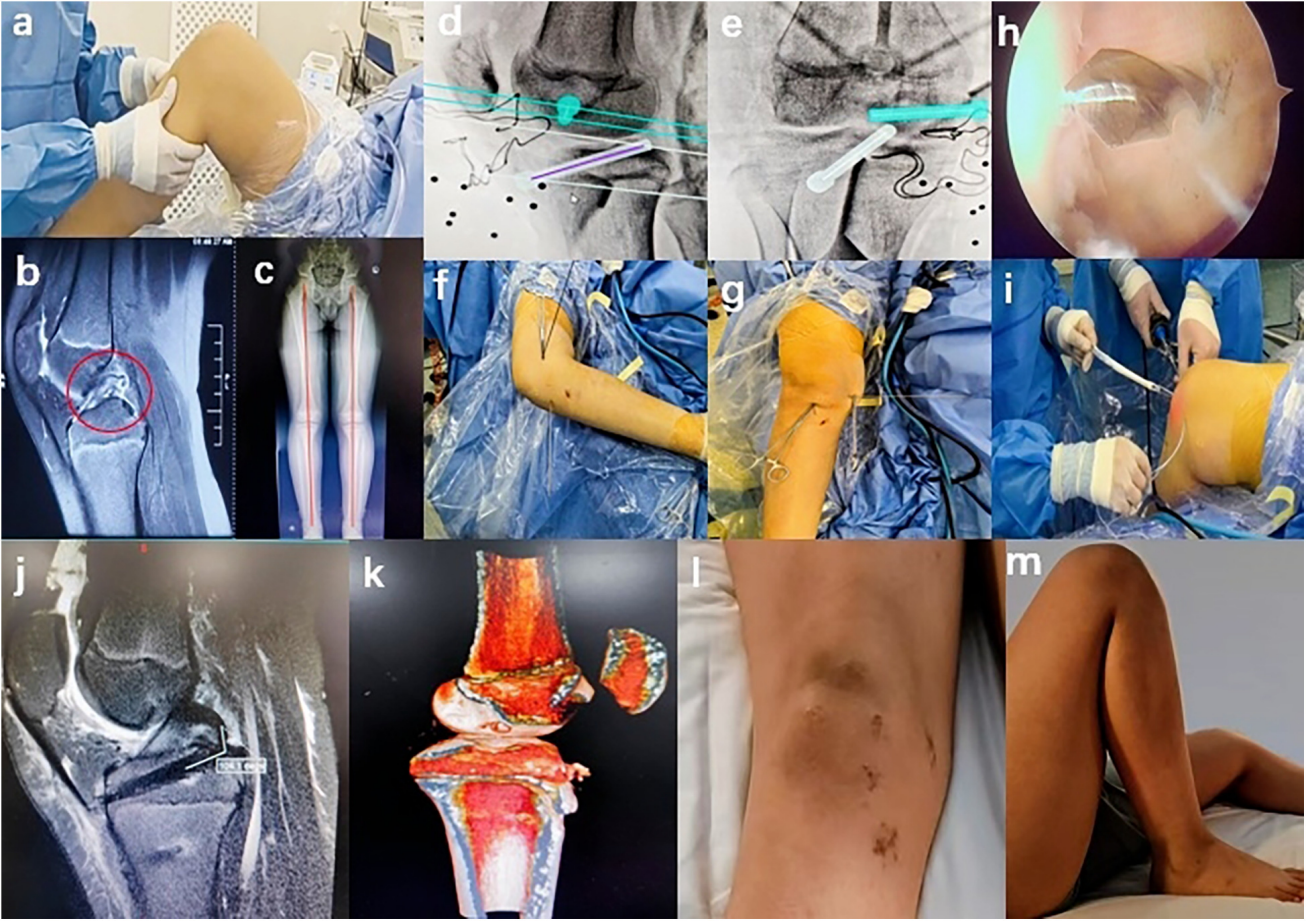
Case presentation: **(a)** positive posterior drawer test, **(b)** preoperative MRI of the right knee revealing discontinuity in the middle and lower segments of the PCL with partial retraction of the ruptured ends, **(c)** preoperative full-length x-ray of both lower extremities indicating no significant abnormalities in their alignment, **(d,e)** blue and white shapes representing the femoral and tibial bone tunnels, respectively, **(f,g)** guide pin positioning, **(h)** arthroscopic observation, **(i)** tendon pulling, **(j)** postoperative MRI showing that the angle between the graft and the tibial tunnel was approximately 104.1°, **(k)** postoperative CT showing that both the femoral and tibial bone tunnels were within the growth plate, **(l)** surgical incision, and **(m)** knee flexion to full angle achieved at week 8.

Upon physical examination, the patient exhibited a positive posterior drawer test (Figure 1a), a positive posterior sag test, and a positive step-off sign. Magnetic resonance imaging (MRI) of the right knee revealed a discontinuity in the PCL (Figure 1b). X-ray imaging showed no significant abnormalities in the alignment of the lower limbs (Figure 1c). The patient was admitted with a diagnosis of PCL injury. The surgeon took into account that the patient was concurrently experiencing a positive influenza A virus infection, accompanied by severe infection symptoms. We instructed the patient to wear lower limb braces for fixation, perform daily ankle pump exercises, elevate the affected limb, and apply ice packs for cold compress to reduce swelling; additionally, we also prescribed oral antiviral drugs to control the infection.

Twenty days later, the patient's symptoms of infection were under control. With informed consent from the patient and approval from the medical ethics committee of the hospital, we proceeded with an arthroscopic all-epiphyseal PCLR with remnant preservation, assisted by the TiRobot surgical system.

### Surgical technique

In this case, the surgery was successfully performed by a highly experienced chief doctor certified in robotic surgery, and the TiRobot system (Beijing TINAVI Medical Technology Co., Ltd.) was used. This advanced system is specifically engineered for precise, minimally invasive orthopedic procedures and comprises a robotic arm, an optical tracking system, and a primary control console. The surgical process was generally segmented into three phases: “Registration,” “Planning,” and “Execution.” Initially, the surgeon securely affixed a tracker to the bone adjacent to the surgical site, after which the engineer performed the “Registration” procedure for the orthopedic surgical robot. Following the transmission of the C-arm scan data of the surgical target area to the computer navigation system within the main console, the engineer collaborated with the surgeon to design the bone tunnel (Figures 1d,e). The engineer then transmitted the planned bone tunnel configuration as a command to the robotic arm via the main console. Subsequently, the robotic arm accurately delineated the bone tunnel externally using the sleeve at its terminus. The surgeon then utilized the hollow sleeve system tool to execute the preprogrammed drilling of the bone tunnel. Furthermore, the navigation system provided continuous real-time optical monitoring and tracking throughout the entire surgical procedure, ensuring that the actual trajectory of the bone tunnel drilling precisely matched the predesigned virtual path.

We first established routine anteromedial and anterolateral arthroscopic portals to thoroughly explore the whole knee joint, assess the tension and integrity of the ACL and PCL, and identify any intra-articular concomitant injuries. A posteromedial approach was then implemented to meticulously debride the posterior tibial attachment of the PCL while preserving a portion of the PCL tibial remnant and appropriately releasing posterior capsular adhesions. Afterward, we selected and harvested the ipsilateral gracilis and semitendinosus tendons, folded them into four strands, braided, and sutured them for subsequent use. After securely affixing the tracer to the distal femur, the engineer adjusted the C-arm position and configured the parameters to obtain standard anteroposterior and lateral radiographic views of the knee joint under fluoroscopy. Initially, we determined the exit point of the intra-articular bone tunnel, then designed the positions of the femoral and tibial bone tunnels, and inputted the data into the robotic system. Under robotic arm-assisted positioning, the surgeon inserted the guide pin, verified its position intraoperatively using fluoroscopy (Figures 1f,g), and confirmed the accuracy of the guide pin placement through arthroscopic examination (Figure 1h). Next, we installed the guide on the guide pin and constructed the femoral and tibial bone tunnels sequentially. We then utilized the prepared traction line to draw the braided tendon into the femoral and tibial bone tunnels (Figure 1i). Finally, we secured the femoral end using an EndoButton (ENDOBUTTON CL ULTRA Fixation Device with a 20-mm continuous loop suture, Smith & Nephew Inc.) and the tibial end with a Versalok suture anchor (DePuy Mitek). Finally, we confirmed that the anterior and posterior drawer tests were negative, conducted a subsequent arthroscopic examination, and observed that the graft position and trajectory were optimal, with satisfactory graft tension upon probe examination.

### Postoperative course

**Figure 2:**
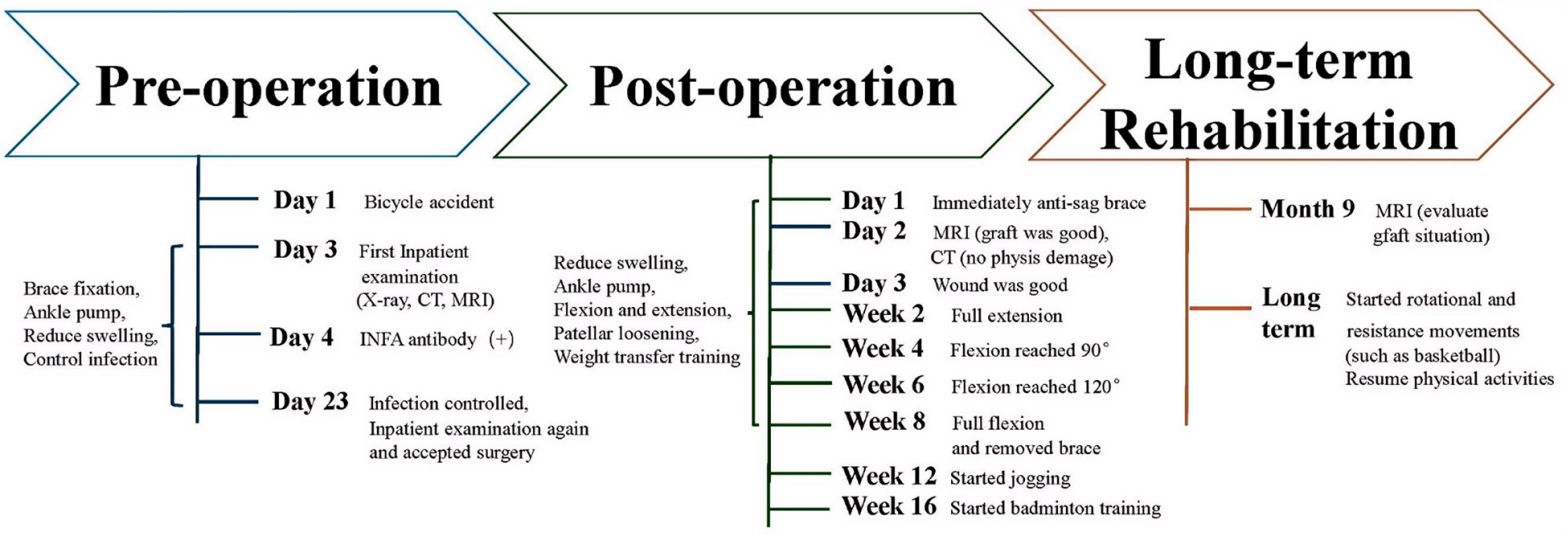
Therapeutic timeline. The treatment process is presented as a timeline starting from the patient's first admission, divided into three phases: preoperation, postoperation, and long-term rehabilitation. On the right side of each phase, time nodes and the patient's condition are indicated, while on the left side, conservative treatments and rehabilitation interventions are indicated. INFA, influenza A virus.

In this case, the surgery lasted 110 min, with four x-ray exposures and one guide pin insertion, without the need for positional correction. Immediately postoperation, an anti-sag brace was applied for fixation, along with compression bandaging using a cotton pad. An MRI review on postoperative day 2 indicated that the graft was securely fixed, with an angle of approximately 104.1° between the graft and the tibial bone tunnel (Figure 1j). Computed tomography three-dimensional reconstruction showed that both the femoral and tibial bone tunnels were within the growth plate (Figure 1k). On day 3, the cotton pads were removed and the dressing was changed; the wound showed no redness or exudate. Knee extension and flexion exercises were initiated. Sutures were removed after week 2, revealing good wound healing (Figure 1l) and complete extension of the affected limb. All rehabilitation training, including passive knee flexion and extension, strength training, and patellar loosening, began on day 3. The strength training within 4 weeks mainly focused on straight leg raises in all directions, multiangle quadriceps exercises, and other long contractions. By week 4, knee flexion reached 90°; by week 6, knee flexion reached 120°; and by week 8, a full range of motion was achieved (Figure 1m). At week 12, the muscle strength of the affected lower limb was over 85% of the healthy side, allowing the patient to start jogging, and by week 16, the patient had resumed badminton training. The rehabilitation training mainly focused on neuromuscular control training and muscle strength enhancement, with the aim of enabling the patient to resume physical activities (Figure 2). At the last follow-up, the patient's knee function had significantly improved, with the International Knee Documentation Committee (IKDC) score improving from 43.68 preoperatively to 82.76 and the Lysholm score increasing from 46 preoperatively to 95.

## Discussion

Currently, treatment strategies for PAPs with PCL injuries exhibit considerable variability, primarily due to factors such as injury severity, patient age, activity level, and the presence of concomitant injuries during treatment plan formulation. Sørensen et al. (2) reported, in a 50-month average follow-up of six children who underwent surgery, that one child developed a leg length discrepancy of 16 mm.

Conversely, many scholars (1, 6, 9, 11) have advocated for surgical treatments. They considered that conservative management may not effectively avert the progression of PCL injuries, potentially resulting in irreversible meniscal damage, increased cartilage degeneration, and a marked decline in overall knee stability. These outcomes not only heightened the likelihood of necessitating surgical intervention in the future but also substantially diminished the probability of patients returning to sports and resuming daily activities. Rupp et al. (4) indicated that adolescents with PCL tears accompanied by meniscal injuries experienced diminished joint stability and exacerbated meniscal damage following conservative treatment. Shah et al. (11) conducted entirely transphyseal arthroscopic surgery on three pediatric patients with PCL injuries and monitored their progress for up to 7 and 9 years, respectively. Kocher et al. (6), through a comparative analysis of 14 surgically treated patients and 11 conservatively managed patients, found no significant differences in functional scores between the two cohorts, and notably, none of the surgically treated patients experienced growth arrest.

Concurrently, various researchers have demonstrated that physeal sparing techniques can also yield satisfactory outcomes while preserving normal growth and development in PAPs with PCL injuries. Wegmann et al. (1) followed nine children who underwent partial transphyseal arthroscopic surgery for up to 70 months and observed that, while the functional scores of the affected knees were lower than those of the contralateral healthy knees, none of the patients exhibited growth disturbances. Bovid et al. (7) documented a case of partial physeal plate PCL reconstruction in an 8-year-old boy, where the femoral tunnel was placed within the epiphysis and the tibial tunnel partially traversed the physeal plate. By the third month postoperatively, the patient achieved 135° of knee flexion and resumed all favored sports, including baseball, by the ninth month. He et al. (8) described a 5-year-old girl who underwent complete physeal suture augmentation fixation for a proximal PCL rupture, achieving satisfactory outcomes. Similarly, Liu et al. (9, 12) reported 13 cases in which guide pins were inserted under fluoroscopic guidance to create bone tunnels that entirely circumvented the physis; in these cases, the femoral tunnels were placed within the epiphysis, and the tibial tunnels were placed outside the physis, thereby minimizing the risk of physeal injury. Their average follow-up period of 25.2 months revealed significant improvements in knee function across all pediatric patients, with no occurrences of growth arrest or angular deformities.

In PCLR for PAPs, meticulous emphasis is placed on anatomical precision, comprehensive biomechanical restoration, and the establishment of optimal healing conditions because these elements are intrinsically linked to the long-term functional recovery and overall quality of life of patients (13–16). The case study presented in this article introduces an innovative approach using the TiRobot system, synergistically integrated with meticulous joint-preserving reconstruction under all-epiphyseal arthroscopy, thereby heralding a new era in PCLR. The core advantages of this approach are outlined as follows:

(1) *Avoidance of physeal damage:* The TiRobot, with its high-precision navigation system and three-dimensional planning capabilities, enables accurate preoperative planning of optimal paths for femoral and tibial bone tunnels, ensuring that each step of the procedure is as close as possible to the ideal anatomical reconstruction position. In addition, during PCLR, this approach maximized the protection of immature growth plates, thereby reducing the risk of growth arrest or angular deformities caused by physeal injuries. Furthermore, TiRobot significantly reduced the frequency of intraoperative x-ray fluoroscopy, effectively protecting patients and medical staff from unnecessary radiation exposure and creating a safer and healthier surgical environment (10, 13).
(2) *Reduction of the “killer turn” effect*: During PCLR, the graft exits the tibial tunnel and advances toward the femoral attachment, forming an angle of less than 90° at the tunnel exit. This configuration led to graft abrasion, thinning, and subsequent loss of tension—a phenomenon commonly referred to as the “killer turn” effect in conventional surgical techniques. To circumvent this problem, some researchers eliminated the use of the tibial tunnel and introduced the Inlay technique, which involved directly affixing the bone–patellar tendon–bone graft to the tibial side within a bone trough (17, 18). Theoretically, this approach allowed the tibial side of the graft to more closely match its anatomical configuration, promoted osseous healing within the posterior bone trough, and prevented graft abrasion while accommodating thicker tendon grafts. However, a significant drawback of this technique was the requirement for a vertical incision at the posterior aspect of the popliteal fossa, which posed a risk to the surrounding vascular and neural structures and consequently increased the likelihood of perioperative complications (17, 18). Similarly, other researchers (19, 20) advocated for an all-inside preservation reconstruction method for the PCL, utilizing a posterior medial double-portal approach under arthroscopy. This technique obviates the need for a posterolateral portal, thereby minimizing the risk of injury to posterior vascular and neural structures, while the additional posterior medial auxiliary approach facilitates enhanced visualization of the PCL's distal attachment and the preservation of its remnants, which reduces the “killer turn” effect. Nonetheless, this technique inevitably impacts the proximal tibial physis to some degree. In PAPs, precise robotic guidance and positioning allow for the avoidance of epiphyseal damage. This method maximally preserves the remnant tissue of PCL's distal attachment and prevents mechanical friction between the graft and the bone tunnel, thereby mitigating the “killer turn” effect.
(3) *Minimization of the risk of injury to surrounding tissues:* The approach significantly mitigates the risk of surgical trauma and complications by obviating the need for a posterior–lateral auxiliary portal, which is customary in conventional adult procedures. Utilizing robotic assistance, the likelihood of the guide pin breaching the posterior tibial cortex was substantially reduced, thereby minimizing potential harm to critical vascular and nerve structures, especially the common peroneal nerve in the posterior popliteal region, thus ensuring surgical safety. Furthermore, the frequency of punctures and guide pin insertion during the procedure was markedly diminished, which reduced interference with surrounding tissues, shortened both the duration of the surgery and tourniquet application, and facilitated faster recovery with fewer complications. In addition, preserving the PCL remnant was invaluable for fostering tendon–bone healing and proprioceptive recovery. This was particularly advantageous for PAPs, as it resulted in a recovery that more closely approximated normal physiological conditions, thereby providing robust support for their return to daily activities and sports.

Moreover, the application of TiRobot has greatly facilitated the standardization and repeatability of surgical procedures. This high level of standardization not only increased the success rate of surgeries but also shortened the learning curve, providing a platform for young surgeons to learn and master advanced techniques. In addition, TiRobot aligns with the advanced concepts of minimally invasive surgery in sports medicine and arthroscopy. During the postoperative recovery phase, the minimally invasive nature of robotic-assisted surgery offers PAPs more convenience and advantages. Minimal surgical trauma and favorable wound healing conditions allow patients to begin rehabilitation training earlier, thus accelerating the recovery of muscle strength and joint flexibility. Furthermore, good tendon–bone healing provides patients with a more stable knee joint and reduces the risk of repeat surgeries due to ligament loosening or rupture.

## Limitations

This study has several limitations. First, as an emerging technology, it remains in the nascent stages of clinical application. The sample size was limited, and the lack of accumulated clinical data undermined the generalizability and reliability of the findings. Further validation is required to establish the maturity and stability of this technology. Second, there was a dearth of long-term postoperative follow-up data because of the poor economic and travel conditions. Such data are indispensable for evaluating surgical outcomes, the incidence of complications, and the stability and functional recovery following ligament reconstruction. In the absence of these data, a comprehensive understanding of the long-term safety and efficacy of this technology remains elusive. We originally planned to conduct an MRI re-evaluation of ligament healing between 9 months and 1 year after the operation and then gradually enable the patient to resume other rotational and resistance movements (such as basketball). However, the patient was lost to follow-up after the 6th month due to personal reasons. However, in the case of this patient, the follow-up results at the end of the 6th month were satisfactory. Third, as advanced medical devices, orthopedic surgical robots entail considerable acquisition, maintenance, and operational costs, which limit their deployment and promotion in primary healthcare settings and economically disadvantaged regions. Furthermore, the technical expertise required to proficiently operate these robots is currently scarce, which may impede their accessibility and restrict their ability to meet the needs of a diverse patient population. Finally, although orthopedic surgical robots offer significant advantages in precision and safety, they are not without technical limitations. For instance, in cases involving complex pathology and unique anatomical structures, the operational capabilities of the robot may be restricted.

## Conclusion

In conclusion, arthroscopic all-epiphyseal PCLR with remnant preservation assisted by TiRobot offers numerous significant advantages—such as prevention of physeal injury, preservation of the PCL remnant, reduction of the “killer turn” effect, and minimization of trauma to adjacent blood vessels and nerves—without physeal damage. However, this technique still faces certain limitations and challenges in practical implementation. Future research endeavors should concentrate on increasing sample sizes, extending long-term follow-up studies, reducing technical costs, and improving accessibility to further advance the development and application of this technology.

## Data Availability

The original contributions presented in the study are included in the article/Supplementary Material, further inquiries can be directed to the corresponding authors.
